# High-Content Lithium Aluminum Titanium Phosphate-Based Composite Solid Electrolyte with Poly(ionic liquid) Binder

**DOI:** 10.3390/polym14071274

**Published:** 2022-03-22

**Authors:** Fujie Yang, Qingfeng Liu, Wenfei Xie, Pu Xie, Jingqi Shang, Xugang Shu

**Affiliations:** 1 GD HPPC Lab, College Chemistry and Chemical Engineering, Zhongkai University of Agriculture and Engineering, Guangzhou 510275, China; liuqingfeng1023@163.com (Q.L.); honmameyiko@163.com (W.X.); shangjq919@163.com (J.S.); 2 School of Chemistry, Sun Yat-Sen University, Guangzhou 510275, China; xie.pu@bngrp.com

**Keywords:** lithium aluminum titanium phosphate, poly(ionic liquid), solid-state electrolyte, ionic conductivity, hardness

## Abstract

Solid electrolytes have been regarded as the most promising electrolyte materials for the next generation of flexible electronic devices due to their excellent safety and machinability. Herein, composite solid electrolytes (CSE) with “polymer in ceramic” are prepared by using lithium aluminum titanium phosphate (LATP) as a matrix and modified poly(ionic liquid) as a binder. The results revealed that adding a poly(ionic liquid)-based binder not only endowed good flexibility for solid electrolytes, but also significantly improved the ionic conductivity of the electrolytes. When the content of LATP in the CSE was 50 wt.%, the electrolyte obtained the highest ionic conductivity (1.2 × 10^−3^ S·cm^−1^), which was one order of magnitude higher than that of the pristine LATP. Finally, this study also characterized the compression resistance of the composite solid-state electrolyte by testing the Vickers hardness, and the results showed that the hardness of the composite solid-state electrolyte can reach 0.9 ± 0.1 gf/mm^2^ at a LATP content of 50 wt.%.

## 1. Introduction

In recent years, the rapid development of the lithium-ion battery has greatly facilitated the portable electronics and electric vehicles markets. Furthermore, it efficiently reduces the consumption of fossil fuels and carbon dioxide emissions, thus alleviating the environmental crisis [1,2,3]. However, current commercial Li-ion batteries are mainly assembled with organic liquid electrolytes, which are leaky, flammable, and volatile, leading to severe security risks [4,5,6]. To address the above issues, active ceramic-based solid electrolytes have been designed and fabricated for increasing the security of Li-ion batteries [7], such as garnet-type lithium lanthanum zirconium oxide [8], LISICON-type lithium-ion conductor [9], NASICON-type lithium aluminum titanium phosphate [10], and perovskite-type lithium lanthanum titanium oxide [11]. Although these active ceramics have excellent bulk ionic conductivity, the grain, grain boundaries, and their interfaces also exist in the electrolyte films, which dramatically decrease the total conductivity [12,13,14]. Therefore, how to achieve a high total conductivity is still an important role for these active ceramic electrolytes. Among these active ceramics, the lithium aluminum titanium phosphate (LATP) with NASICON structure reveals a significant advantage for material synthesis and practical commercialization, considering the relatively low prices of raw materials and high ionic conductivities (10^−4^∼10^−3^) [7,15,16]. Moreover, the high stability of LATP against H_2_O/O_2_ allows the material preparation and battery assembly to proceed in the ambient air, further reducing the manufacturing cost [7]. Although the LATP solid electrolyte material possesses good performance, the interfacial compatibility and fabrication feasibility are greatly sacrificed due to the structural brittleness and rigidity, which limits the development of this type of electrolyte [17,18,19].

Numerous studies have proven that the combination of active ceramic materials and organic polymers can effectively improve the contact interfaces of inorganic solid electrolytes and significantly increase the ionic conductivity of active ceramic-based electrolytes to a certain extent [20,21,22,23,24,25]. The enhanced ionic conductivity is mainly because the size, morphology, and volume fraction of active ceramics can be appropriately adjusted for decreasing the crystallinity of the polymer matrix and forming the efficiency networks of ionic conduction, leading to providing more ion transport pathways [26,27,28]. These kinds of composite electrolytes have attracted people’s attention due to the advantages of high ionic conductivity, good flexibility, mechanical properties, easy processing, and good interface contact [29]. For instance, Jin and co-workers used polymethyl methacrylate (PMMA) to functionalize the surface of LATP, and then used the interaction between PMMA and polyvinylidene fluoride (PVDF) molecules to prepare the LATP@PMMA-PVDF composite solid electrolyte, which shows a higher ionic conductivity (1.76 × 10^−3^ S·cm^−1^). Nonetheless, the ceramic content in the composite solid electrolyte is usually less than 40 wt.% due to the poor contact between internal phase interfaces, which results in the undesirable agglomeration of ceramic particles after passing the percolation threshold [30]. It should not benefit from maximizing the high ionic conductivity of active ceramics.

Poly(ionic liquid) is an emerging family of polymer electrolytes which takes advantage of both the mechanical durability of polymers and the electrochemical properties of ionic liquid [31,32,33]. In this paper, in order to improve the ion transport performance of a high-content (≥50 wt.%) LATP composite solid electrolyte, a high-content LATP copolymer electrolyte is prepared by using LATP as the matrix and cooperating with the modified imidazolium poly(ionic liquid) as the binder. The prepared LATP composite solid electrolyte reveals high ionic conductivity, good film-forming processability, and high hardness. It is hoped that the strategy of the as-prepared LATP copolymer electrolytes could offer a new insight into the development of the advanced solid electrolytes with outstanding performance.

## 2. Materials and Methods

### 2.1. Materials

1-vinylimidazole (99%), chloropropane (99%), butyl acrylate (99%), and 2,2-azobis(2-methylpropionitrile) (AIBN, 98%) were purchased from Aladdin Reagent (Shanghai, China). 1-ethyl-3-methylphenidate bifluorosulamide salt (LITFSI, 99%), aluminum nitrate nonahydrate nine hydrate (Al(NO_3_)_3_·9H_2_O, 99%), lithium nitrate (LiNO_3_, 99%), and toluene (99%) were obtained from Innochem (Beijing, China). Lithium bis(trifluoromethane sulfonyl)imide (LITFSI, 99%) was provided by Saiwei Electronic Materials Co., Ltd. (Zhuhai, China).

### 2.2. Synthesis of LATP Composite Electrolytes

LATP was fabricated according to the published literature [34]. Briefly, the Li_1.3_Al_0.3_Ti_1.7_(PO_4_)_3_ powders were synthesized via a modified Pechini sol-gel method, followed by a calcination process. For the sol-gel process, a precursor solution was obtained by dissolving stoichiometric amounts of LiNO_3_, Al(NO_3_)_3_·9H_2_O, and NH_4_H_2_(PO_4_)_3_ in a mixed solvent of Ti(C_4_H_9_O)_4_ and nitric acid, and 10 wt.% excess LiNO_3_ was added to compensate for Li^+^ volatilization during the calcination process. Citric acid and lactic acid as complexing agents were introduced for a homogeneous dispersion of metal cations. The solution was evaporated under 80 °C to obtain a gel precursor, followed by further heat treatment in a vacuum drying oven at 200 °C for 50 h. Then, obtained LATP powders were calcined in an aluminum crucible at 850 °C for 5 h and then ground with a zirconia ball for 10 h to reduce the particle size. Finally, the LATP powders were cold-pressed and sintered at 900 °C for 5 h.

The modified poly(ionic liquid) was prepared according to the following synthetic route. Firstly, 1-vinylimidazole and chloropropane were added to toluene at a molar ratio of 1:2, and stirred at 50 °C for 3 h. Subsequently, butyl acrylate was added in the same molar ratio compared with 1-vinylimidazole, and AIBN was added and used as the initiator. After reacting for 48 h in an oil bath at 60 °C, the resulting precipitate was washed and filtered with anhydrous ether, and extracted with ether for 48 h. After that, the above product was subjected to anion replacement, added to 10 mL of 0.4 mol/L LiTFSI ethanol solution, and stirred for 24 h, and then a small amount of deionized water was added dropwise to make the precipitate separate out. Finally, the precipitate was washed 5 times with deionized water and dried under vacuum for 24 h to obtain imidazolium-based poly(ionic liquid) (denoted as P(IL-BA)TFSI).

**Scheme 1:**
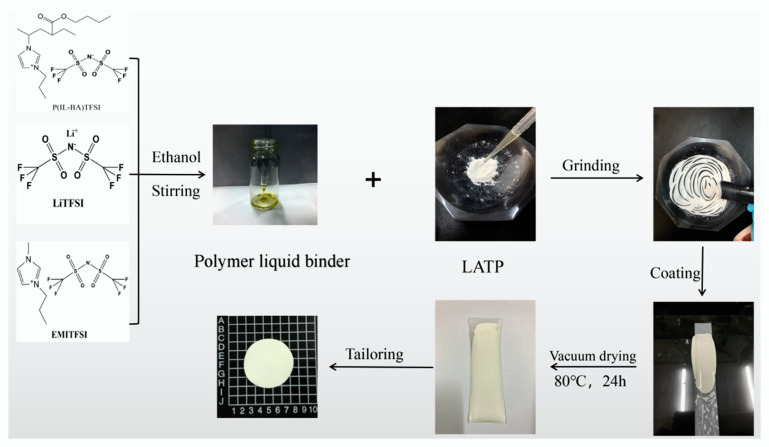
Preparation process of the composite solid electrolyte.

A uniformly dispersed poly(ionic liquid)-based adhesive was fabricated after mixing the P(IL-BA)TFSI, LiTFSI, and 1-ethyl-3-methylphenidate bifluorosulamide in 10 mL of anhydrous ethanol at a mass ratio of 2:1:1. Then, composite solid electrolytes with 50, 60, 70, 80, and 90 wt.% of LATP were prepared, respectively. A certain amount of LATP powder was ground in the agate mortar for 10 min, with the gradual addition of the modified poly(ionic liquid) base adhesive. After grinding until the mixture becomes viscous, the composites were coated on the polytetrafluoroethylene film and then transferred into a vacuum drying oven to dry at 80 °C for 24 h. After that, LATP/poly(ionic liquid) composite solid electrolytes were obtained. The specific operations are shown in Scheme 1.

### 2.3. Characterizations

A Fourier infrared spectrometer (FT−IR, Bruker Tensor 27, Bruker, Germany) and X-ray diffractometer (XRD, Rigaku DMAX 2200, Rigaku, Japan) were used to characterize the characteristic groups and crystal structure of electrolyte materials. The microscopic morphology and element distribution of the sample were examined through a scanning electron microscope (SEM, Hitachi S−4800, HITACHI, Japan) equipped with an X-ray energy spectrum. A TA Q50 series thermal weight loss analyzer (TG, TA Q50, TA Instrument, New Castle, DE, USA) and TA Q10 series differential scanning calorimeter (DSC, TA Q10, TA Instrument, New Castle, DE, USA) were applied to test the thermal stability and glass transition temperature of the samples, respectively.

### 2.4. Electrochemical Measurements

For testing the electrochemical properties, the prepared composite solid electrolytes were tailored into a circle with diameters of 16 mm and placed between two stainless-steel electrodes to fabricate a symmetrical coin-cell. The thickness of the circle sample was about 0.075 cm. The electrodes with diameters of 15.8 mm were supplied by Canrd New Energy Technology Co., Ltd. (Guangdong, China). The ionic conductivity of the composite solid electrolytes was calculated by electrochemical impedance spectroscopy (EIS), with the frequency ranging from 1 MHz to 0.1 Hz on a CHI660E electrochemical station. Subsequently, using the open circuit voltage of the battery as the bias voltage, the ionic conductivity in the temperature range of 30–80 °C was used to obtain the corresponding activation energy. The calculation formula is [35]:

(1) $$\sigma_{b}=\frac{l}{R_{b}\cdot S}$$

where *σ_b_* is the bulk ion conductivity, *l* is the thickness, *R_b_* is the bulk resistance, and *S* is the area of the electrode–electrolyte contact (1.96 cm^2^). The ionic conductivity, *σ,* and activation energy (*E*_a_) at a certain temperature were good following the formula:

(2) $$\sigma T=A_{T}\exp(\frac{-E_{a}}{kT})$$

where *A_T_* is the front factor, *T* is the test temperature, and *k* is the Boltzmann constant. In order to minimize the cracks of the sample, the load was 0.098 and 0.245 N, the indentation time was 10 s, and the composite electrolyte was tested for micro-Vickers hardness. Vickers hardness, *H_v_*, can be calculated by the formula [36]:

(3) $$H_{v}=\frac{1854.4P}{d^{2}}$$

In the formula, *H_v_* is the Vickers hardness, *P* is the applied load, and *d* is the length of the diagonal of the Vickers indentation.

## 3. Results

### 3.1. Structural Characterization of the Composite Electrolyte Materials

**Figure 1:**
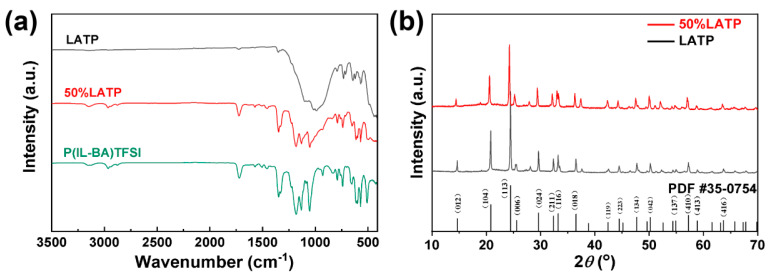
FT−IR spectra (**a**) and XRD patterns (**b**) of composite electrolytes.

Firstly, in order to explore whether there is an interaction between LATP and the prepared poly(ionic liquid), we used FT-IR to characterize the characteristic functional groups inside the 50% LATP-based composite solid electrolyte, LATP, and poly(ionic liquid)-based binder samples, as illustrated in Figure 1a. It can be seen that the characteristic peak of the PO_4_ group was around 990 cm^−1^, and the peak between 410 and 700 cm^−1^ represents the bond outside the PO_4_ structure formed by aluminum, titanium, and lithium. The low-intensity peaks at 631–536 cm^−1^ belong to the Ti-O stretching vibration of the TiO^6−^ octahedron, which overlaps the bending vibration of the PO_4_^3-^ tetrahedron [37]. The broad bands at 500–400 cm^−1^ resulted from the structural distortion in the rhombohedral unit cell due to Ti^4+^ substitution by Al^3+^ [38]. Compared with the LATP sample, the composite solid electrolyte containing 50 wt.% LATP has not only the characteristic functional groups of LATP, but also the characteristic functional groups of the poly(ionic liquid) structure. For example, 1722 cm^−1^ was the C=O vibration peak on the poly(ionic liquid), near 3150 cm^−1^ was the C–H stretching vibration peak on the unsaturated carbon of the imidazole ring, and 2936–2965 cm^−1^ was the C–H stretching vibration peak on the saturated carbon. Near 930 cm^−1^ was the stretching vibration peak of C-F [39], 1181 cm^−1^ was the C–O–C stretching vibration peak, near 1230 cm^−1^ was the C–N stretching vibration peak on the imidazole ring, and the imidazole ring at 1571 cm^−1^ due to skeleton vibration, and the C=N vibration peak on the imidazole ring was near 1453 cm^−1^. Moreover, the displacement and shape of the characteristic peak of LATP did not change significantly after adding poly(ionic liquid). This shows that the poly(ionic liquid) mechanically binds LATP particles and does not affect the chemical environment of LATP.

XRD was further used to analyze the internal crystalline states of the prepared LATP-based composite solid electrolyte, as shown in Figure 1b. It can be seen that the prepared LATP matches well with the standard pattern (PDF #35-0754), indicating that a relatively pure and crystallized LATP active ceramic was obtained. By comparing the pristine LATP sample, the characteristic diffraction peaks of the 50% LATP composite solid electrolyte have no obvious changes in the peak shape and peak position. The result indicates that the addition of P(IL-BA)TFSI did not affect the intrinsic crystalline state of LATP during the prepared process of the composite solid electrolyte. 

**Figure 2:**
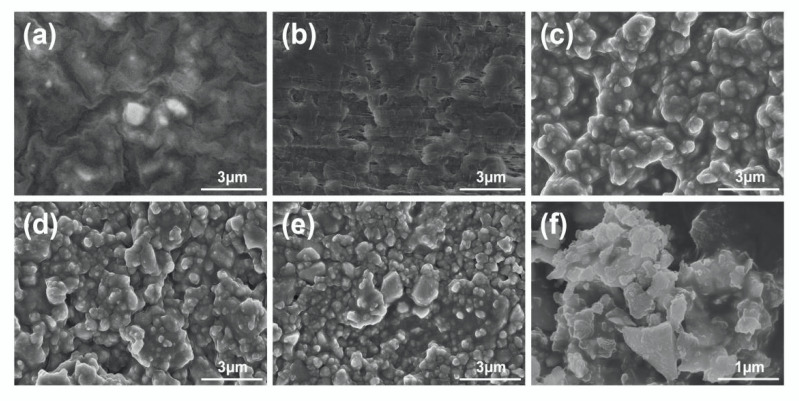
SEM images of composite electrolytes with different LATP content. (**a**–**f**) Represent 50% LATP, 60% LATP, 70% LATP, 80% LATP, and 90% LATP composite electrolytes, and pristine LATP electrolyte, respectively.

The distribution of LATP particles in the composite solid-state electrolytes was characterized by SEM, as shown in Figure 2. The SEM results also revealed the different microstructures in various LATP content of the composite solid electrolytes. Compared with the pristine LATP (Figure 2f), the LATP particle dispersion was more uniform after adding the poly(ionic liquid) binder. With the increase of LATP content, the aggregation phenomenon of the particles became more and more clear. Among them, the 50% LATP composite electrolyte and the 60 wt.% LATP composite electrolyte hardly observed particle aggregation on the surface of the samples. When the LATP content was ≥70 wt.%, numerous particles adhered to the polymer binders could be observed in the composite electrolytes. It indicates that the prepared poly(ionic liquid) binder possesses a good adhesion for the LATP particles, which is conducive to obtain the LATP-based solid electrolytes with high-content LATP.

**Figure 3:**
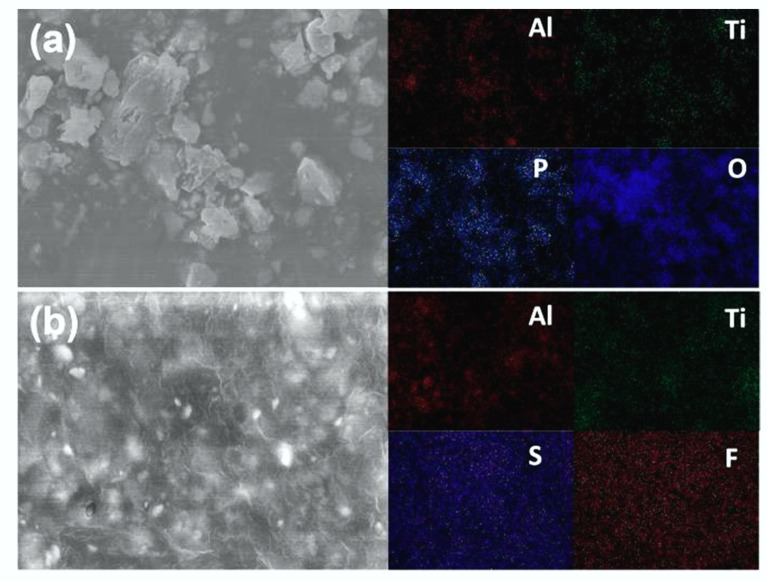
EDS mapping images of LATP (**a**) and 50% LATP composite electrolyte (**b**).

Furthermore, the feature element distribution in the 50% LATP composite solid electrolyte was also explored by using the SEM-EDS mapping technique, as shown in Figure 3. It can be clearly seen from Figure 3a that the main constituent elements (Al, Ti, P, O) of the prepared LATP particles were uniformly distributed on the corresponding particles. Similar to the pristine LATP, the preceding elements belonging to LATP were clearly detected in the 50% LATP composite solid electrolyte. Moreover, the S and F elements derived from P(IL-BA)TFSI were also observed in the composite electrolyte. Those results indicate that the compatibility between the LATP and the poly(ionic liquid) binder is very high, and the former particles can be dispersed uniformly in the latter by physical adhesion.

### 3.2. Ion-Conduction Properties of Composite Electrolytes

**Figure 4:**
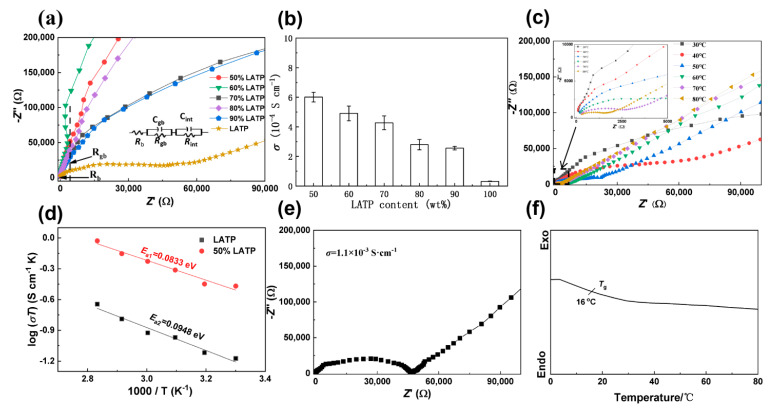
EIS curves (**a**) (inset equivalent circuit, *R*_b_: bulk resistance, *R*_gb_: grain boundary resistance, *R*_int_: interfacial resistance, C_gb_ and C_int_: double layer capacitance) and relevant ionic conductivities (**b**) of composite electrolytes with different content of LATP. EIS curves of LATP−based composite electrolytes at different temperatures (**c**). Arrhenius curves of LATP−based composite electrolytes and pristine LATP electrolytes (**d**). EIS (**e**) and DSC (**f**) curves of the poly(ionic liquid) electrolyte.

The influences of LATP content on the bulk impedance and ionic conductivity of the composite solid electrolyte were analyzed by electrochemical impedance spectroscopy (EIS) technology. Figure 4a shows the EIS curve of the LATP-based composite electrolytes. The bulk resistance (*R*_b_) of various electrolytes can be obtained from the EIS curve, and the corresponding ionic conductivity (*σ*_b_) is calculated from *R*_b_ (see Figure 4b). Besides, to further test the grain boundary conductivities (*σ*_gb_) of the different content LATP-based composite solid electrolytes, the impedance spectrum was fitted with the equivalent circuit, in which *R*_b_ represents bulk resistance, *R*_gb_ represents the grain boundary resistance of the composite electrolyte, and *R*_int_ represents the interfacial resistance. The corresponding detailed values are shown in Table 1. The *R*_b_ value of the composite electrolyte gradually increased with the increase of LATP content. When the LATP content was 50 wt.%, the *R*_b_ value of the composite electrolyte was the smallest, and its corresponding *σ*_b_ value was the largest, reaching 1.2 × 10^−3^ S·cm^−1^. The *σ*_b_ value of a single LATP solid electrolyte tested under the same conditions was only 5.8 × 10^−5^ S·cm^−1^. This phenomenon indicates that the addition of a poly(ionic liquid)-based binder can significantly improve the ion conductivity of the LATP-based solid electrolyte. However, it was found that when the LATP content was less than 50%, too much poly(ionic liquid) binder would lead to over-viscosity of the composite electrolyte film, which was not conducive to film formation. Therefore, it is appropriate to control the LATP content above 50 wt.%. The value of *R*_gb_ at room temperature was 4922 Ω for the 50% LATP-based composite solid electrolyte, corresponding to the *σ*_gb_ of 7.78 × 10^−6^ S·cm^−1^. Compared with the pure LATP electrolyte, the possible reason for the lower *σ*_gb_ of the LATP-based composite solid electrolyte is that the space charge layers exist in the interfaces between the LATP particles and the modified poly(ionic liquid) [40], which is resistive to Li^+^-ion conduction. Although LATP-based composite solid electrolytes have lower *σ*_gb_ values, the total ionic conductivities (including *σ*_b_ and *σ*_gb_) are still obviously higher than those of the pure LATP due to the significant improvement of *σ*_b_ values.

In order to further clarify the transport kinetics of ions in the composite electrolytes, the ionic conductivities of the 50% LATP composite electrolyte at different temperatures were studied by EIS measurement (see Figure 4c). It can be seen that the *R*_b_ values were decreased and the *σ* values were increased with the gradually increasing temperature. The ionic conductivity–temperature curves of the LATP composite electrolytes were reasonably fitted by the Arrhenius behavior. The *E*_a_ values of LATP and 50% LATP electrolytes were 9.1 kJ mol^−1^ (0.0948 eV) and 8.0 kJ mol^−1^ (0.0833 eV), respectively. Therefore, the *E*_a_ values of LATP composite electrolytes were obviously lower than those of the LATP electrolytes. The result indicates that the ion transport of the composite solid electrolyte is less affected by temperature, and the ions are mainly conducted through the “jumping” of ions to adjacent holes [41]. It is worth mentioning that the EIS curve of the single poly(ionic liquid) electrolyte was also tested in this work (see Figure 4e). The corresponding ionic conductivity of the poly(ionic liquid) electrolyte was 1.1 × 10^−3^ S·cm^−1^. The higher ionic conductivity of the poly(ionic liquid) electrolyte is not only related to the fact that the polymer structure can dissociate lithium salt, but also closely related to its lower glass transition temperature (*T*_g_ = 16 °C, see Figure 4f).

### 3.3. Hardness and Thermal Stability of Composite Electrolytes

**Figure 5:**
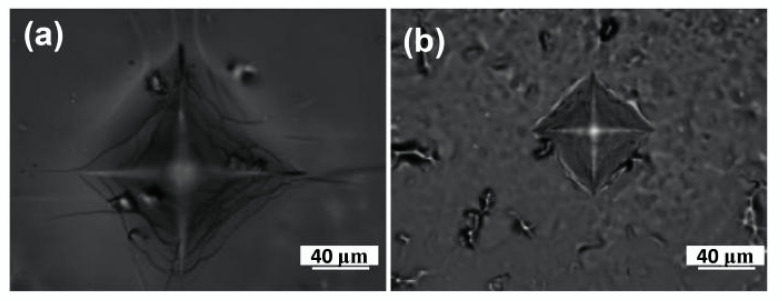
Scanning electron images of Vickers indentation of 50% LATP (**a**) and 90% LATP (**b**) composite electrolytes.

The solid electrolyte not only plays the role of a bridge for the transmission of ions, but also blocks the positive and negative electrodes to prevent internal short circuits in the battery. Therefore, its hardness and thermal stability directly affect the safety performance of the battery. Figure 5 shows that the indentation size of the 50% LATP composite electrolyte was between 126 and 155 μm, and the indentation size of the 90% LATP composite electrolyte was between 66 and 85 μm. In the micro-nano scale, the Vickers hardness value of the material increases with the decrease of the indentation size [42]. It can be seen from Table 2 that as the content of LATP increased, the Vickers hardness of the composite electrolyte increased, which indicates that the increase of the poly(ionic liquid) binder will cause the brittleness of the electrolyte to decrease and the toughness of the electrolyte to increase.

**Figure 6:**
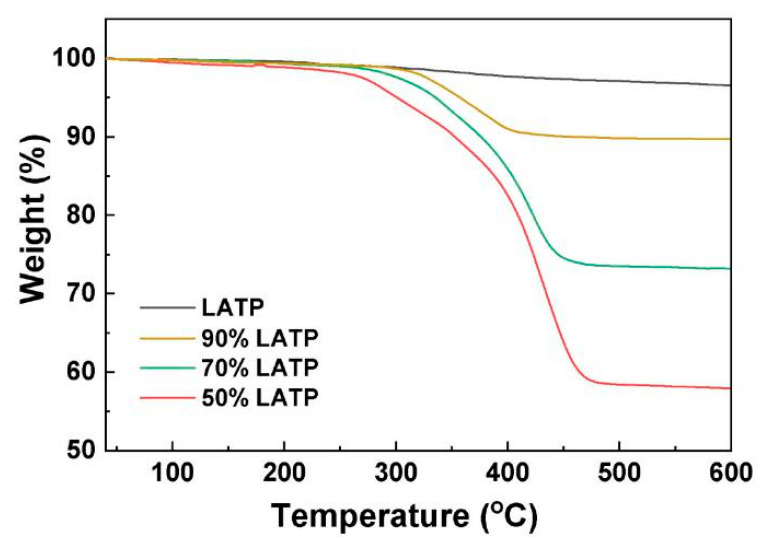
Thermogravimetric analysis curves of composite solid electrolytes with different content of LATP.

TGA analysis was conducted to investigate the thermal stability of the electrolyte membranes; as presented in Figure 6, the initial decomposition of all the composite electrolytes was at about 250 °C. At the same time, as the content of poly(ionic liquid) binder increased, the thermal decomposition products increased. This shows that the composite electrolyte film can heat up to 250 °C and has high thermal stability.

## 4. Conclusions

In summary, a high-content LATP-based composite solid-state electrolyte was prepared. A series of structural characterizations proved that there is no interaction between the compound of LATP and P(IL-BA)TFSI poly(ionic liquid), and the addition of the poly(ionic liquid) binder helped to improve the film-forming processability of the composite solid electrolyte. The ionic conductivity of the 50% LATP-based composite electrolyte was more than one order of magnitude higher compared to that of the LATP solid electrolyte, and was slightly higher than that of the poly(ionic liquid). Moreover, the activation energy of the composite electrolyte was obviously lower than that of the LATP solid electrolyte. These results indicate that the addition of poly(ionic liquid) can significantly improve the ionic conductivity of the composite electrolyte, possibly due to the space charge layers between them that facilitate ion transport. In addition, the prepared LATP composite electrolyte possesses high hardness and excellent thermal stability. Therefore, the prepared LATP-based composite electrolyte has high ionic conductivity, good film-forming performance, and high safety. The high-content active ceramic-based composite electrolyte with excellent performance is expected to provide a new idea for the development of high-performance solid electrolytes for next-generation flexible electrochemical devices.

## Figures and Tables

**Table 1:** Bulk impedance, grain boundary resistance, and ionic conductivities of composite solid electrolytes with different content of LATP.

	50% LATP	60% LATP	70% LATP	80% LATP	90% LATP	LATP
*R*_b_/Ω	31.2	46.77	57.4	71.98	91.05	158
*R*_gb_/Ω	4922	4262	4911	3335	1752	600.1
*R*_int_/Ω	5.54 × 10^5^	7.96 × 10^5^	2.63 × 10^5^	4.45 × 10^5^	1.1 × 10^6^	4.38 × 10^4^
*σ*_b_/S·cm^−1^	1.2 × 10^−3^	8.3 × 10^−4^	6.6 × 10^−4^	5.3 × 10^−4^	4.2 × 10^−4^	5.8 × 10^−5^
*σ*_gb_/S·cm^−1^	7.78 × 10^−6^	8.98 × 10^−6^	7.80 × 10^−6^	1.15 × 10^−5^	2.18 × 10^−5^	1.53 × 10^−5^
*σ*_int_/S cm^−1^	6.91 × 10^−8^	3.64 × 10^−8^	1.45 × 10^−7^	8.6 × 10^−8^	3.48 × 10^−8^	2.09 × 10^−8^

**Table 2:** Hardness test of LATP-based composite solid electrolytes.

Sample	Load/Holding Time	Hardness, *H_v_* (gf/mm^2^)
50% LATP	10 g f (0.098 N)/10 s	0.9 ± 0.1
90% LATP	25 g f (0.245 N)/10 s	8.6 ± 1.2

## Data Availability

The data obtained in this study are available within the article.
